# The Breastfeeding Self-Efficacy Scale-Short Form (BSES-SF): a validation study in Iranian mothers

**DOI:** 10.1186/s13104-019-4656-7

**Published:** 2019-09-23

**Authors:** Payam Amini, Reza Omani-Samani, Mahdi Sepidarkish, Amir Almasi-Hashiani, Mostafa Hosseini, Saman Maroufizadeh

**Affiliations:** 1 Department of Biostatistics and Epidemiology, School of Public Health, Ahvaz Jundishapur University of Medical Sciences, Ahvaz, Iran; 2 Department of Medical Ethics and Law, Reproductive Biomedicine Research Center, Royan Institute for Reproductive Biomedicine, ACECR, Tehran, Iran; 3 Department of Biostatistics and Epidemiology, Babol University of Medical Sciences, Babol, Iran; 4 Department of Epidemiology, School of Health, Arak University of Medical Sciences, Arak, Iran; 5 Department of Epidemiology and Biostatistics, School of Public Health, Tehran University of Medical Sciences, Tehran, Iran; 6 School of Nursing and Midwifery, Guilan University of Medical Sciences, Rasht, Iran

**Keywords:** Breastfeeding, Self-efficacy, Reliability, Validity, Iran

## Abstract

**Objective:**

The Breastfeeding Self-Efficacy Scale-Short Form (BSES-SF) is a widely used instrument that measures breastfeeding self-efficacy. This study aimed to examine the reliability and validity of the Persian version of BSES-SF in Iranian mothers.

**Results:**

The English version of BSES-SF was translated into Persian using the standard forward–backward translation procedure. No changes (i.e., neither delete nor rephrase the items) were made to the BSES-SF items. The mean BSES-SF total score was 50.80 ± 8.91. The Cronbach’s alpha coefficient for internal consistency for the BSES-SF was 0.910. The confirmatory factor analysis results provided evidence for unidimensionality of the scale (χ^2^/df = 4.42; CFI = 0.96; NFI = 0.95; IFI = 0.96; RMSEA = 0.095 and SRMR = 0.054). The divergent validity of the BSES-SF was proved via a significant negative correlation with scores of the Edinburgh Postnatal Depression Scale (r = − 0.273, P < 0.001). In sum, the Persian version of the BSES-SF is a reliable and valid instrument for measuring breastfeeding self-efficacy in Iranian mothers.

*Trial registration number* This was a cross-sectional study (not clinical trial).

## Introduction

Breastfeeding is the optimal method of feeding and nurturing infants [1]. It has short- and long-term advantages for the baby and the mother. As such, the World Health Organization and American Academy of Pediatrics recommend exclusive breastfeeding for the first 6 months of the infant’s life, with continued breastfeeding for at least 1 or 2 years [2, 3]. Despite these benefits and recommendations, not all mothers initiate and continue to breastfeed. Breastfeeding self-efficacy is a woman’s confidence in her ability to breastfeed [4] and is a salient variable in the initiation and duration of breastfeeding [5].

The Breastfeeding Self-Efficacy Scale (BSES) was developed by Dennis and Faux in 1999 to assess breast-feeding confidence [6]. This scale is a 33-item, self-administered instrument, where items are preceded by the phrase “I can always” and scored on a 5-point Likert scale ranging from 1 (not at all confident) to 5 (always confident). As such, total scores can range from 33 to 165, with higher scores reflecting greater levels of breastfeeding self-efficacy. In 2003, Dennis [7] revised the BSES from 33 to 14 items and renamed it the BSES-Short Form (BSES-SF). The theoretical framework is the same as the BSES. A considerable amount of reliability and validity evidence supports its use as a global measure of breastfeeding self-efficacy. The reliability and validity of this instrument was satisfactory in USA [8], Canada [9–11], Brazil [12, 13], UK [14], Spain [15], Italy [16], Sweden [17], Poland [18], Croatia [19], Portugal [20], Turkey [21], China [22], Japan [23], Malaysia [9], and Hong Kong [24].

Due to the declining breastfeeding rates in Iran, it was considered essential to validate a Persian version of the BSES-SF and use it as one component in a strategy for improving the breastfeeding rate. Therefore, this study aimed to evaluate the reliability and validity of the BSES-SF as a measure of breast-feeding confidence among Iranian mothers.

## Main text

### Methods

#### Participants and study design

In this cross-sectional study, breastfeeding mothers referring to a health center for neonatal vaccination in Tehran, Iran were invited to participate in the study. We collected data between July and September 2017. The sample size was determined using the rule of thumb suggested in the literature. Norusis [25] indicated that the sample size should be at least 300 cases for factor analysis studies. Besides, Comrey and Lee [26] thought that the sample size of 300 is suitable for factor analysis studies. Breastfeeding mothers were eligible to take part in this study if they were: (a) married, (b) were 16 years of age or older, (c) willingness to participate in the study, and (d) able to read and write Persian. In total, 379 mothers agreed to take part and fill out the questionnaires completely.

#### Translation of the BSES-SF into Persian

The English-language version of the BSES-SF was translated into Persian using the standard forward–backward translation procedure. First, items and response choices were translated into Persian independently by two native Persian speakers, both of whom were fluent in English. The two forward translations were then combined into one version by the research team. This forward translation was translated back into English by a professional translator and compared to the original scale. Additional small changes were performed to ensure that the Persian version did not differ from the original English version. No changes (i.e., neither delete nor rephrase the items) were made to the BSES-SF items.

#### Measures

##### Demographic information and obstetrics factors of the mother and infant

Basic demographic and obstetric characteristics of the mother and infant included: mother’s age, level of education, occupation, duration of the marriage, parity, delivery mode, type of pregnancy, infant age, and infant sex.

##### Breastfeeding Self-Efficacy Scale-Short Form (BSES-SF)

The BSES-SF is a 14-item self-administered instrument derived from the original 33-item BSES that measures breastfeeding confidence [7]. All items are preceded by the phrase ‘‘I can always’’ and rated on a 5-point Likert scale, ranging from 1 (not at all confident) to 5 (always confident). Total scores range from 14 to 70, with higher scores reflecting more significant levels of breastfeeding self-efficacy.

##### Edinburgh Postnatal Depression Scale (EPDS)

The EPDS is a commonly used self-administered instrument that measures postnatal depression [27]. Respondents rate items on a 4-point Likert scale, ranging from 0 to 3. Total scores range from 0 to 30, with higher scores reflecting greater postnatal depression. The Persian version of EPDS has shown sound psychometric properties in Iranian populations [28]. In the current study, the Cronbach’s alpha coefficient of the EPDS was 0.790.

##### Perceived Stress Scale-10 Item (PSS-10)

The PSS-10 is a commonly used self-administered instrument derived from the original 14 item PSS (PSS-14) that measures perceived stress [29]. The PSS-10 measures global stress or “the degree to which situations in one’s life are appraised as stressful.” Respondents rate items on a 5-point Likert scale, ranging from 0 (never) to 4 (very often). Total scores range from 0 to 40, with higher scores reflecting more significant stress. The Persian version of PSS-10 has been validated among infertile patients and adults with asthma [30, 31]. In the current study, the Cronbach’s alpha coefficient of the PSS-10 was 0.825.

#### Data analysis

To examine the unidimensionality of the BSES-SF, we performed a confirmatory factor analysis using maximum likelihood estimation method. Model fit was evaluated using several goodness-of-fit indices including the Chi-square/degree of freedom (χ^2^/df), the comparative fit index (CFI), incremental fit index (IFI), the normed fit index (NFI), the root mean square error of approximation (RMSEA), and the standardized root mean square residual (SRMR). Values of χ^2^/df < 5, CFI, IFI, and NFI > 0.90, and RMSEA and SRMR < 0.08 indicate an adequate fit of the model to the data [32–35]. Cronbach’s alpha, inter-item correlation, and corrected-item total correlation were calculated to assess the internal consistency of the scale. The convergent validity of the BSES-SF was evaluated by calculating Pearson correlation coefficients between the BSES-SF scores and measures of the EPDS and PSS-10. To assess floor and ceiling effects, we calculated the percentage of mothers achieving the lowest (1) and highest (5) possible scores for items. Floor and ceiling effects > 20% are considered to be significant [36].

Data analyses were conducted using SPSS version 16 for Windows (SPSS Inc., Chicago, IL, USA) and LISREL 8.80 (Scientific Software International, Inc., Lincolnwood, IL, USA).

### Results

#### Mothers characteristics

The mean maternal age of the mothers was 30.13 years (SD = 5.81; range 16–45). Of the participants, 29.8% were university-educated, 12.9% were employed, 57.6% were primiparous. CS was reported for 72.7% of the sample (of which 65.3% responded planned CS (Table 1).

**Table 1:** Demographic and obstetrics characteristics of the mothers (n = 379)

	Mean ± SD or n (%)
Mother’s age (years)	30.16 ± 5.76
Educational level	
Primary/secondary	267 (70.4)
University	112 (29.6)
Occupation	
Housewife	330 (87.1)
Employed	57 (12.9)
Duration of marriage (years)	8.00 ± 5.43
Parity	
Primiparous	159 (42.0)
Multiparous	220 (58.0)
Delivery mode	
Vaginal	104 (27.4)
Cesarean	275 (72.6)
Type of pregnancy	
Wanted	322 (85.0)
Unwanted	57 (15.0)
Infant age (months)	103.77 ± 128.03
Infant sex	
Male	191 (50.4)
Female	188 (49.6)

*SD* standard deviation

#### Descriptive statistics of the BSES-SF

The BSES-SF items and their mean and standard deviation (SD) are presented in Table 2. Question 5 (‘Manage the breastfeeding situation to my satisfaction’) had the lowest mean item score (mean = 3.00, SD = 1.06). The mean total score was 50.80 (SD = 8.91; range 14–69). There was no floor or ceiling effect for any of the 14 items and the overall rating of BSES-SF.

**Table 2:** Item wording, descriptive statistics and internal consistency of the BSES-SF

	Mean	SD	Corrected item-total correlation	Alpha if item deleted	Floor effect (‘1’%)	Ceiling effect (‘5’%)
1. Determine that my baby is getting enough milk	3.42	0.85	0.342	0.913	2.1	4.7
2. Successfully cope with breastfeeding like I have with other challenging tasks	3.72	0.84	0.607	0.905	1.1	13.2
3. Breastfeed my baby without using formula as a supplement	3.63	1.06	0.632	0.904	6.9	15.3
4. Ensure that my baby is properly latched on for the whole feeding	3.74	0.92	0.601	0.905	3.7	15.0
5. Manage the breastfeeding situation to my satisfaction	3.00	1.06	0.270	0.919	6.3	6.9
6. Manage to breastfeed even if my baby is crying	3.51	0.98	0.633	0.903	3.7	9.2
7. Keep wanting to breastfeed	3.68	0.94	0.722	0.900	5.5	11.6
8. Comfortably breastfeed with my family members present	3.54	0.99	0.547	0.907	5.5	9.5
9. Be satisfied with my breastfeeding experience	3.75	0.97	0.768	0.898	5.8	15.0
10. Deal with the fact that breastfeeding can be time-consuming	3.68	0.96	0.658	0.902	4.7	13.5
11. Finish feeding my baby on one breast before switching to the other breast	3.81	0.91	0.743	0.899	4.0	15.6
12. Continue to breastfeed my baby for every feeding	3.78	0.88	0.777	0.898	2.6	16.4
13. Manage to keep up with my baby’s breastfeeding demands	3.82	0.81	0.757	0.900	2.4	14.0
14. Tell when my baby is finished breastfeeding	3.72	0.91	0.677	0.902	4.5	11.9
Total BSES-SF score	0.00	0.00	–	–	1.1	0

*SD* standard deviation

#### Internal consistency

The Cronbach’s alpha coefficient for the BSES-SF was 0.910 and was not increased by more than 0.10 if any item was deleted. All corrected item-total correlations were within acceptable range except for Item 5 (“Manage the breastfeeding situation to my satisfaction”). This item had a corrected item-total correlation of 0.270. The mean corrected item-total correlation for the 14 items was 0.624. The mean interitem correlation was 0.428, with values ranging from 0.068 to 0.752. According to the inter-item correlation matrix, Item 1 and Item 5 had low association with other items.

#### Construct validity

To test the unidimensionality of the BSES-SF, the CFA was carried out. The goodness-of-fit indices indicated that the model did not fit the data well (χ^2^/df = 5.82; CFI = 0.95; NFI = 0.94; IFI = 0.95; RMSEA = 0.113 and SRMR = 0.064). Examination of the modification indices recommended allowing covariance between Item 1 and Item 2 as well as between Item 3 and Item 4 (see Additional file 1: Figure S1). A better fit was obtained by considering aforementioned covariances (χ^2^/df = 4.42; CFI = 0.96; NFI = 0.95; IFI = 0.96; RMSEA = 0.095 and SRMR = 0.054). As shown in Additional file 1: Figure S1, all factor loadings were greater than 0.50, except for item 1 and 5, which were slightly lower.

#### Divergent validity

As expected, there was a significant negative correlation between BSES-SF and EPDS scores (r = − 0.273, P < 0.001), indicating satisfactory divergent validity. However, contrary to our expectation, there was no significant relationship between BSES-SF and PSS-10 scores (r = − 0.068, P = 0.189).

#### Breastfeeding self-efficacy and demographic and infant variables

As presented in Table 3, according to Pearson correlation, there were no significant correlation between BSES-SF and mother’s age, the duration of marriage, and infant age (all P > 0.05). Independent t-test also shows that education, occupation, parity, delivery mode, type of pregnancy, and infant sex were not related to BSES-SF scores (all P > 0.05).

**Table 3:** Relationship of BSES-SF scores with demographic characteristics (n = 379)

	r or mean ± SD	P
Mother’s age (years)	0.001	0.997
Duration of marriage (years)	0.055	0.284
Infant age (months)	− 0.041	0.426
Educational level		0.575
Primary/secondary	50.63 ± 8.94	
University	51.20 ± 8.88	
Occupation		0.641
Housewife	50.88 ± 8.87	
Employed	50.24 ± 9.28	
Parity		0.934
Primiparous	50.75 ± 8.87	
Multiparous	50.83 ± 8.96	
Delivery mode		0.221
Vaginal	51.56 ± 6.32	
Cesarean	50.51 ± 9.71	
Type of pregnancy		0.671
Wanted	50.72 ± 9.01	
Unwanted	51.26 ± 8.39	
Infant sex		0.399
Male	51.18 ± 9.01	
Female	50.41 ± 8.82	

*r* Pearson correlation, *SD* standard deviation

### Discussion

This study examined the reliability and validity of the BSES-SF in a sample of Iranian mothers. The internal consistency of the BSES-SF was proved as both Cronbach’s alpha and corrected item-total correlations were high. Similar results have been found in other studies validating the BSES-SF [8, 10, 15, 19, 22, 24, 37]. The CFA result yielded empirical support for the unidimensional conception of the BSES-SF. The unidimensionality of the BSES-SF has been documented via CFA in Chinese [22], and Hong Kong Chinese [24] versions and our results are consistent with them. Besides, this structure has been reported in previous studies using exploratory factor analysis approach [8, 10, 15, 19, 37]. However, the internal consistency and CFA findings suggest that some modifications for item 1 and 5 might be needed in the scale to yield better internal consistency and high factor loading. Consequently, minor modifications in item wording or deleting the items may be necessary when administrating this scale to breastfeeding mothers. One the other hand, a cross-cultural difference might contribute to these results in our study. Further multicenter validation studies with large sample size in populations with different cultural backgrounds are needed to confirm our findings.

Evidence of divergent validity of the BSES-SF was proved by high negative correlation with the EPDS. This result is in line with two validation study conducted in Brazil [12] and Italy [16]. Previous studies also reported that the BSES-SF scores were considerably related to other measures of breastfeeding self-efficacy or theoretically related concepts including breastfeeding attitude questionnaire [10], general self-efficacy scale, stress management self-efficacy scale [15], and sense of coherence scale [19].

The mean of BSES-SF was 50.80 (SD = 8.91), which is lower than what was reported in the original BSES-SF and studies conducted in Brazil [12], Sweden [17], Turkey [21], Poland [18], Croatia [19], but higher than what was reported in China [22], Japan [23], and Hong Kong [24].

Similar to our study, studies using the BSES-SF in other countries did not find any relationship between breastfeeding self-efficacy and mother’s age, education, or type of delivery [8, 10, 12, 14, 15, 17, 37]. Contrary to previous research [5, 14, 38] and breastfeeding self-efficacy theory [39] demonstrating higher levels of breastfeeding self-efficacy among multiparous women, there was no difference between primiparous and multiparous on breastfeeding self-efficacy in this study.

In conclusion, in light of satisfactory reliability and validity, the BSES-SF is a quick and straightforward instrument for the assessment of breastfeeding self-efficacy among Iranian mothers. The findings reported in the current study are following those derived in other validation studies. However, the BSES-SF might be improved if items one and five were removed from the scale. These items had low loadings on the breastfeeding self-efficacy factor, which decreased their internal consistency.

## Limitations

There were two limitations in this study that should be noted. First, test–retest reliability was not evaluated. Second, we could not able to follow-up the baby’s feeding and therefore, to examine the predictive validity.

## Supplementary information

**Additional file 1: Figure S1.** Confirmatory factor analysis of the one-factor model of BSES-SF.

## Data Availability

The datasets used and/or analyzed during the current study are available from the corresponding author on reasonable request.

## Abbreviations

SD: standard deviation
BSES: Breastfeeding Self-Efficacy Scale
BSES-SF: Breastfeeding Self-Efficacy Scale-Short Form
EPDS: Edinburgh Postnatal Depression Scale
PSS-10: Perceived Stress Scale-10 Item
CFA: confirmatory factor analysis
CFI: Comparative Fit Index
NFI: Normed Fit Index
IFI: Incremental Fit Index
RMSEA: root mean square error of approximation
SRMR: standardized root mean square residual
