# Supplementary figures and images for: The Breastfeeding Self-Efficacy Scale-Short Form (BSES-SF): a validation study in Iranian mothers
Source: BMC Res Notes. 2019 Sep 23;12:622. doi: 10.1186/s13104-019-4656-7 (PMC6757403; doi:10.1186/s13104-019-4656-7)

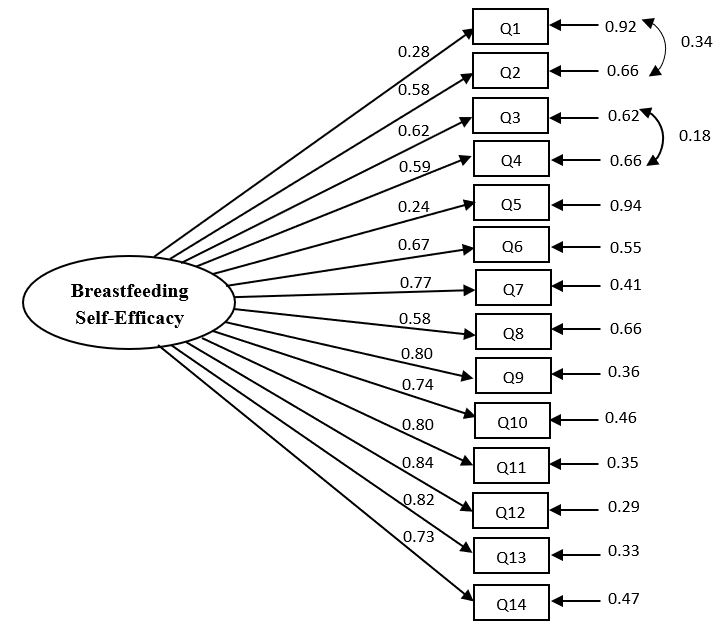

Supplement: Supplementary file 1 — Additional file 1: Figure S1. Confirmatory factor analysis of the one-factor model of BSES-SF. [file 13104_2019_4656_MOESM1_ESM.jpg]
